# The effects of university students’ fragmented reading on cognitive development in the new media age: evidence from Chinese higher education

**DOI:** 10.7717/peerj.13861

**Published:** 2022-08-23

**Authors:** Wei Liu, Heng Huang, Atif Saleem, Zhongping Zhao

**Affiliations:** 1 College of Teacher Education, College of Education and Human Development, Zhejiang Normal University, Jinhua, China; 2 School of Teacher Education, Shaoxing University, Shaoxing, China

**Keywords:** Cognitive development, Fragmented reading, University students, Media learning, Chinese higher education

## Abstract

**Introduction:**

The impacts of fragmented reading have been felt on a huge scale during the new media age. An increasingly fast-paced society and a corresponding drop in theoretical reading have affected reading literacy and cognitive development across communities—and among university students in particular. This study sought to identify the components of fragmented reading and cognitive development and investigate the former’s effects on the latter among university students in the new media age.

**Methods:**

Paper-based and electronic surveys were used to gather demographic and related-reading data from undergraduates at six Chinese universities in early 2021. After testing the data from 916 samples for reliability and validity, descriptive statistics were obtained and path analysis was undertaken using structural equation modeling.

**Results:**

The students reported relatively high levels of fragmented reading, particularly in its temporal form. Significant associations were found between the constructs of fragmented reading and cognitive development. Specifically, the fragmentation of content, time, and attention in reading behavior had significant, positive effects on cognitive breadth. However, each of these components was somewhat negatively associated with cognitive depth.

**Conclusions:**

The findings disclosed the dual effects of fragmented reading on the cognitive development of students, opening a new perspective on this debate. As fragmented reading in the new media age grows inexorably, the study highlights the need to utilize its positive effects on cognitive development by integrating and classifying fragmented information into the mental maps of learners.

## Introduction

Reading is an essential human activity that enables people to know the world and learn their inherited culture. Its importance is recognized by UNESCO (United Nations Educational, Scientific, and Cultural Organization), which has promoted the activity since 1995 by designating April 23 as World Reading Day. The recent advent of information technology has meant that paper books are less frequently read than content on various electronic devices (Piaget, 1973; Rose, 2011). Moreover, an increasingly fast-paced society and various psychological factors have led to a growth in fragmented reading, whose features include scattered content, its intermittent nature, and the limited attention span of readers.

According to China’s National Reading Survey, 79.3% of reading in the country now occurs on a screen (Wei & Xu, 2021). This demonstrates the extent of the shift to digital reading, with everyday, fragmented content becoming increasingly pervasive, along with a corresponding drop in theoretical, academic, and systematic reading matter. These claims are supported by the National Reading Survey’s finding that 84.3% of university students regard smartphones and the Internet as their basic channels to obtain daily information. While fragmented reading is now prevalent among university students (Ding, 2019; Gómez & Vallecillo, 2021), reading is a complex process, requiring individuals to deploy multiple aspects of their cognition (Adams, 1990). The act of reading can be conceptualized as a process of upgrading students’ cognitive development, including the breadth and depth of their cognition (Hoover & Gough, 1990). Cognitive development depends on a complex of factors including the medium (paper- or screen-based), choice of text, the absorption of knowledge, and the ability to acquire, think critically about, and reconstruct meaning. Observing the reading activities of university students thus offers a means to measure their level of cognitive development (Floyd et al., 2007). This is particularly important because young adult university students, a valuable human resource for any society, are at a critical developmental stage (Hartshorne & Germine, 2015), yet the widespread practice of fragmented reading among this group may significantly impact their cognitive development.

Existing studies that explore the relationship between different reading behaviors and students’ development assume that on-screen reading is associated with a more fragmented reading style (Delgado & Salmerón, 2021). Students who read intermittently on new media devices demonstrate lower cognitive ability and academic performance than students who read in fixed time using traditional paper books (Wu & Wei, 2017). However, these studies provide insufficient evidence to determine whether this difference results from fragmented reading.

Research seeking to demonstrate the effects of fragmented reading on cognition has provided mixed results. While some studies suggest that fragmented reading may actually improve university students’ reading ability and level (Jiang et al., 2017; Wang et al., 2015), others find that fragmented reading significantly impacts young people’s reading literacy, causing inertia and atomized thinking (Gu, 2018). These conflicting conclusions may arise from methodological differences: numerous prior studies have addressed only a single aspect of fragmented reading and cognitive development (Lau & Ho, 2016), rather than investigated the multiple factors involved. Others have used qualitative methods at an insufficient scale, making findings impossible to generalize (Wang, 2016).

This study identified three dimensions of students’ fragmented reading behavior and deployed cognitive devolvement theory (Barrouillet, 2015) to specify its effects on cognitive breadth and depth. Structural equation modeling (SEM) was used to analyze the survey data collected from six Chinese universities on the research topic. The results aim to provide evidence-based guidance to students whose reading behavior is fragmented in order to refine aspects of their cognitive development.

### Fragmented reading

The term “fragmentation” first appeared in the literature on reading behavior in the 1980s, in connection to postmodernism. Fragmented reading is held to consist of both content-related and temporal aspects, characterized by rapid attentional shifts from one short text to another (Barbiero, 2008; Zhang, 2013). With comprehensive mobile network coverage and the popularity of smart devices in the new media age, fragmented reading is practiced by increasing numbers of students (Mokhtari, Reichard & Gardner, 2009).

Fragmented reading is a recent phenomenon, with studies describing its frequency, location, and purpose (Mokhtari, Reichard & Gardner, 2009), as well as the software used to practice it (Zhao, 2016). The effects of fragmented reading on university students’ learning motivation, learning autonomy, and efficiency have also been discussed (Kua, Armitage & Branch, 2017). However, while previous studies have identified some characteristics of fragmented reading, the components of time, content, and attention have not been adequately distinguished. This conceptual confusion has contributed to the mixed findings reported in the literature to date.

In addition to fragmentation in the dimensions of content and time identified in prior research (Mokhtari, Reichard & Gardner, 2009; Zhao, 2016), the separation and dispersion of reading time and content will weaken the development of readers’ attention to a certain extent (Gu, 2018), further reducing the time allocated to reading texts and attracting the reader to more texts of reduced length (Hayles, 2007). In line with these findings, the present study divides fragmented reading into three dimensions, referring to the fragmentation of content, time, and attention (Wang, 2018).

Specifically, content fragmentation refers to the characteristics of low continuity and high dispersion, manifested in the shorter content of texts (Xie, 2019). Temporal fragmentation refers to the practice of reading in breaks between classes, waiting for buses, etc., to improve the utilization of time and acquire more information (Fan, 2015; Li, 2011). Attentional fragmentation refers to the integration of digital text, pictures, and sound, which stimulate students’ interest through their visual and auditory senses, attracting their attention to a rapid succession of diverse content (Hayles, 2007; Wang & Wang, 2018).

### Cognitive development

First proposed by Piaget (1973), the concept of cognitive development included four stages: the sensorimotor stage, preoperational stage, concrete operational stage, and formal operational stage. Among these, the sensorimotor and preoperational stages refer to the process of receiving information and expanding cognition horizontally, increasing cognitive breadth. The concrete operational and formal operational stages represent the processes of encoding the information received and internalizing it vertically to develop cognitive depth (Fischer, 1980). Thus, human cognitive development proceeds from a horizontal to a vertical direction, from a low to a high level, and from the initial reception of external data and information to its internalization as knowledge and wisdom (Jing & Cheng, 2005). The progressive relationship between cognitive breadth and depth describes how cognition develops.

Gagne’s information processing theory also points out that the size and quantity of information that can be stored in individual short-term memory is highly limited, and information can be stored in long-term memory only after repeated, fine processing, organization, and coding (Gagne, 1968). Thus, short-term memory processes support the expansion of cognitive breadth, while those of long-term memory encourage the growth of cognitive depth.

Most research on this area has focused on students’ cognitive development in early childhood and adolescence (Gagne, 1968; Mungas et al., 2013; Thompson et al., 2019), overlooking the cognitive development of university students and its influence by social factors. While the precise peak age for cognitive performance remains unknown, it is generally thought to decline from the late 30s (Doppelt & Wallace, 1955). Thus, as young or emerging adults, university students are at a “golden age” of cognition, both post-developmental and pre-senescent (Baltes, 1987; Greenwood, 2007). However, the emergence of electronic devices and the resulting transformation of reading methods may have impacted students’ cognitive development, to an uncertain extent.

The literature (Berry, Gruys & Sackett, 2006; Ko-Woon, Young & Hoy-Yong, 2019) tends to focus either on the development of cognitive breadth or its depth. Cognitive breadth refers to the breadth of university students’ vision, the frequency with which they update information, and their agility in searching for knowledge. Cognitive depth refers to the degree of mastery, memory, and integrity of knowledge that students can demonstrate.

### Fragmented reading and cognitive development

The particular significance of reading for students is its role in developing critical thinking and comprehension skills that are foundational to learning in any subject area (Rutherford-Becker & Vanderwood, 2009). Similarly, reading and writing are hugely important in supporting and reflecting the development of an individual’s cognitive system (Kravchenko, 2021). It is thus highly likely that reading style will directly or indirectly affect the cognitive development of students.

However, the findings of research investigating the effects of fragmented reading on cognitive development are contradictory. This may be because they have tended to approach each construct holistically, without examining their particular components in sufficient depth, or because they measured only a single aspect of each area. In terms of the positive effects of fragmented reading on cognition, it was found that mobile reading typical of a fragmented style improved students’ cognitive skills when accompanied by targeted reading instruction (Cheng & Wang, 2021). Fragmented reading also appears to benefit cognitive development by increasing students’ enjoyment of reading (Ding, 2019; Jiang, 2017; Wang et al., 2015), refining their core subject knowledge, and aiding the accumulation of professional knowledge and ability (Gómez & Vallecillo, 2021). It also significantly improves students’ reading ability and provides additional opportunities for practice (Xie, 2019), thereby broadening their horizons (He & Liu, 2018).

However, many other studies indicate that fragmented reading restricts cognitive development. It has been associated with lower levels of abstract thought and a reduced ability to process content in traditional media such as books (Wu & Wei, 2017). Moreover, fragmented reading was found to increase the likelihood of students overlooking auxiliary material (Velázquez, 2011), while the division and dispersion of reading time were found to impact the development of readers’ attention and their ability to integrate knowledge systematically (Gu, 2018).

Moreover, the limited consensus on how fragmented reading impacts cognitive development has been achieved through qualitative methods, with more advanced analytical approaches such as confirmatory factor analysis and structural equation modeling rarely employed (Bennett et al., 2003). Further, earlier research efforts have neither investigated fragmented reading in terms of its constituent parts nor approached cognition in terms of breadth and depth. Most significantly, the current confusion makes it impossible for users of the research (students in particular) to adapt their reading behavior to maximize its effects on their cognitive development.

All these factors prompted this empirical study of the effects of fragmented reading on cognitive development. The research primarily aimed to help students clarify the complex role of fragmented reading and its relevance to their cognitive development, with the hope this might ultimately improve its breadth and depth.

### Research model and hypotheses

The present study sought to extend previous efforts by conducting an SEM analysis of the prevalence of fragmented reading among Chinese university students and its effects on their cognitive development. Based on the findings of previous research, we proposed the following six hypotheses:

H1: Content fragmentation has a positive effect on cognitive breadth.

H2: Temporal fragmentation has a positive effect on cognitive breadth.

H3: Attentional fragmentation has a positive effect on cognitive breadth.

H4: Content fragmentation negatively affects cognitive depth.

H5: Temporal fragmentation negatively affects cognitive depth.

H6: Attentional fragmentation negatively affects cognitive depth.

**Figure 1:**
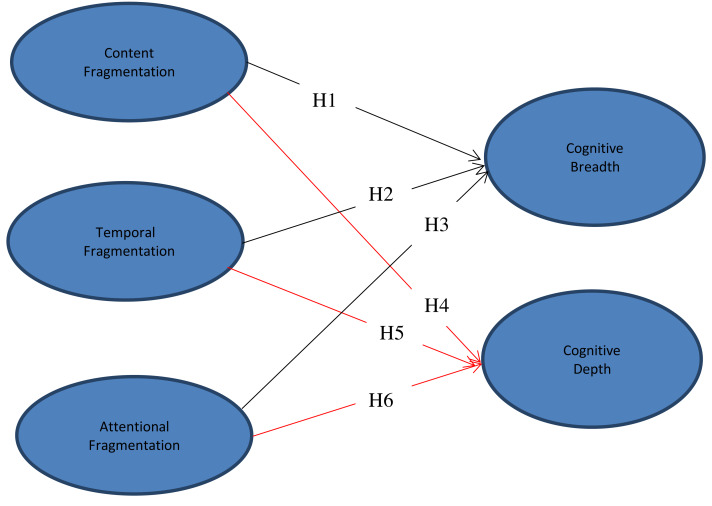
Research model, black color: positive effect; red color: negative effect.

Based on the above hypotheses, the following model (Fig. 1) was constructed:

## Materials & Methods

### Sample and procedure

The above hypotheses were tested by randomly sampling participants from six Chinese universities: Beijing Normal University, Northeast Normal University, Zhejiang Normal University, Liaoning Normal University, Qinghai Normal University, and Yancheng Normal University. A total of 947 questionnaires were distributed *via* post and online in early 2021, of which 916 were completed, an effective response rate of 96.7%. The final sample consisted of males (28.6%) and females (71.4%), with ages ranging from 17 to 31. Approximately 65% of the students were liberal arts majors, with the others (35%) majoring in sciences. Undergraduate students numbered 695 (75.9%), with 221 postgraduates among the participants (24.1%).

### Measures

The cognitive development questionnaire (CDQ) was used in preference to the Cognitive Ability Scale, which has been widely adopted to measure people’s logical thinking, graphical integration, etc. However, we declined to use this instrument as it focuses on specific cognitive abilities. The CDQ, based on the earlier work of Jing & Cheng (2005), was used because it measures cognitive development from the macro perspective. In line with our research interests, we divided the CDQ into two dimensions: cognitive breadth (five items) and cognitive depth (six items). The Fragmented Reading Questionnaire (FRQ) is developed based on the work of Wang (2018); this instrument consisted of 22 items classified into three dimensions: content fragmentation (seven items), temporal fragmentation (five items), and attentional fragmentation (ten items) (see Supplementary, questionnaire). Unless otherwise stated, all responses were recorded on a five-point Likert scale (1 = Strongly disagree; 5 = Strongly agree). Of note, further evidence of the validity of these questionnaires is provided in the confirmatory factor analyses (CFA) described below, and it evidenced the valid use of the measures.

### Statistical analytic procedures

The mean values, standard deviations, and Pearson correlations of all variables were first calculated, followed by path analysis using SEM to examine the magnitude and significance of associations between different variables. To ensure the internal consistency of the constructs, Harman’s single factor test and confirmatory factor analysis were first performed for each question. Maximum likelihood estimation was used to check the model fit. The Chi-square statistic (X2/df), comparative fit index (CFI), normed fit index (NFI), Tucker–Lewis index (TLI), and root mean square error of approximation (RMSEA) were used to evaluate the fit of the research models. The recommended thresholds for each index were X2/df < 3; CFI, NFI, T *L* > 0.90; RMSEA < 0.08 (Hair et al., 2010; Hu & Bentler, 1999). SPSS v.26 and Amos v.26 were used to conduct the statistical analysis.

### Ethical concerns

Ethics committee approval was obtained from Zhejiang Normal University’s institutional review board. The ethical principle of informed consent was upheld: each participant in the questionnaire was informed in advance of what was to be studied, and its possible benefits and impacts. All were informed of their right to withdraw their agreement to participate at any stage before the study was published. Finally, we upheld the right to privacy by preserving the participants’ anonymity at all points in the research process, ensuring that the publication of the research would not result in any conflicts of interest.

## Results

### Construct reliability and validity

Firstly, Harman’s single factor test was conducted to identify common method variance. The explanatory variance of the first common factor was 30%, significantly lower than the 40% threshold, confirming the validity of the instrument used to gather data. Secondly, the reliability and validity of the constructs of content, temporal and attentional fragmentation, as well as cognitive breadth and depth, were tested, and items with chi-square/df over 0.5 were eliminated during CFA (Saleem et al., 2020). Six of the FRQ items measuring content fragmentation were retained, as were four assessing temporal fragmentation, while the section covering attentional fragmentation was unchanged. Two CDQ items were deleted and the remainder were distributed equally between cognitive breadth and depth to ensure the flow of constructs.

Table 1 shows Cronbach’s Alpha values in the range of *α* = 0.70 to 0.79, with 0.7 indicating a high degree of internal consistency among items (Hancock & Mueller, 2013).

**Table 1:** Item validity analyses.

Dimension	Item	FL	AVE	CR	Cronbach’s
1. Content fragmentation	A1–A6	0.62–0.73	0.67	0.75	0.70
2. Temporal fragmentation	B1–B4	0.68–0.80	0.62	0.71	0.72
3. Attentional fragmentation	C1–C10	0.70–0.83	0.64	0.73	0.78
4. Cognitive breadth	D1–D4	0.66–0.81	0.68	0.79	0.73
5. Cognitive depth	E1–E4	0.69–0.78	0.65	0.76	0.79

AMOS software was then used to perform CFA to investigate the reliability and validity of each dimension of the theoretical model. Table 1 demonstrates that the factor loading (FL) values ranged from 0.62 to 0.83, with those recorded in the average variance extracted (AVE) test between 0.62 and 0.68, thus surpassing the threshold of 0.6 for acceptable reliability (Hair et al., 2010). In addition, the composite reliability (CR) value for each of the five dimensions exceeded the conventional threshold of 0.7 (Hair et al., 2010; see Table 1).

### Descriptive statistics and correlations

The mean values, standard deviations, and Pearson correlations for all indicators are presented in Table 2. Among the three dimensions of fragmented reading, temporal fragmentation recorded the highest mean value (*M* = 3.39, SD = 0.50), followed by content fragmentation (*M* = 3.20, SD = 0.26) and then attentional fragmentation (*M* = 3.09, SD = 0.18). Overall, the mean values of each dimension of fragmented reading all exceeded the threshold, M ≥ 3 (Saleem et al., 2020). These results are consistent with previous studies and indicated that fragmented reading is widespread among Chinese university students.

**Table 2:** Descriptive statistics and correlation matrix.

Variable	1	2	3	4	5
1. Content fragmentation	—				
2. Temporal fragmentation	0.63^*^	—			
3. Attentional fragmentation	0.60^**^	0.61^**^	—		
4. Cognitive breadth	0.41^*^	0.50^**^	0.46^**^	—	
5. Cognitive depth	–0.46^**^	–0.38^**^	–0.78^**^	–0.38^*^	—
M	3.20	3.39	3.09	3.37	3.01
SD	0.26	0.50	0.18	0.29	0.35

**Notes.**

* *p* < 0.001.

** *p* < 0.01.

M, arithmetic mean; SD, standard deviation.

In addition, all correlations were found to be statistically significant (see Table 2). Cognitive breadth was positively correlated with the fragmentation of content, time, and attention, with coefficients of 0.41, 0.50, and 0.46, respectively, but cognitive depth had a negative association with each; the strongest correlation was between fragmented attention and cognitive depth (r = 0.78, *p* < 0.01). These results indicated a trend of covariation between the various dimensions of fragmented reading and cognitive development. Structural equation modeling was then used to further explore the relationship between the variables. The correlative relationship was not strong (above 0.85), indicating no obvious multicollinearity (Kline, 2015).

### SEM results

**Figure 2:**
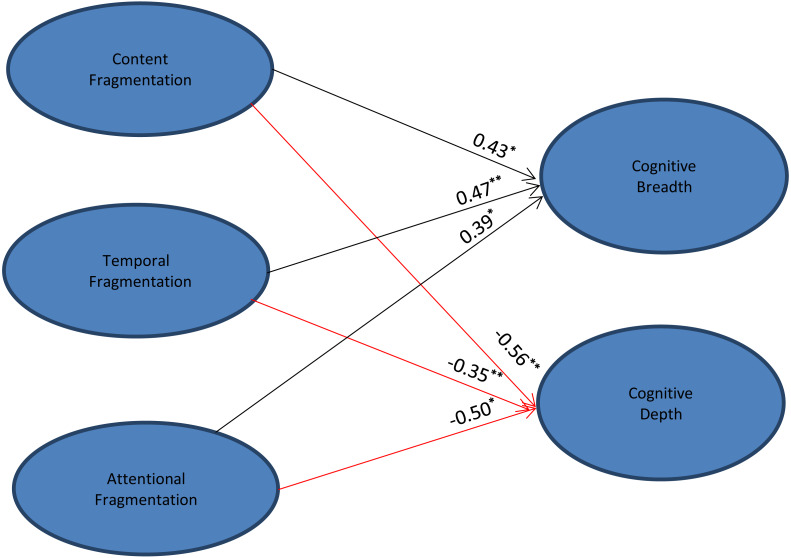
Structural equation modeling (SEM). Significant regression paths; An asterisk (*) indicates *p* < 0.001, two asterisks (**) indicate *p* < 0.014. *β* values are standardized regression coefficients (SRCs), and *n* = 916. Model fit indices: X^2^/df = 2.51, CFI = 0.92, NFI = 0.91, TLI = 0.94, RMSEA = 0.07. Black color: positive effect; red color: negative effect.

Figure 2 displays the final SEM model, which indicated that the structural model was a good fit to the data (X^2^/*df* = 2.51, CFI = 0.92, NFI = 0.91, TLI = 0.94, and RMSEA = 0.07). As expected, parameter estimates showed that each component of fragmented reading exerted a statistically significant effect on cognitive breadth and depth. Fragmentation of content (*β* = 0.43, *p* < 0.001), time (*β* = 0.47, *p* < 0.01) and attention (*β* = 0.39, *p* < 0.001) had positive associations with cognitive breadth; temporal fragmentation exerted the strongest effect of the three on the scale. This indicates that higher levels of (particularly) temporal fragmentation in students’ reading behaviors predicted greater cognitive breadth. In contrast, cognitive depth was negatively associated with all three factors of fragmented reading: content (*β* = −0.56, *p* < 0.01), time (*β* = −0.35, *p* < 0.01), and attention (*β* = −0.50, *p* < 0.001). In other words, students tended to achieve lower levels of cognitive depth when their reading behaviors were more fragmented.

## Discussion

This paper describes one of the first empirical, quantitative investigations into the effect of fragmented reading on students’ cognitive development in Chinese institutions of higher education. As such, it makes a significant contribution to elucidating the relationship between fragmented reading and cognitive development.

The findings not only demonstrate how widespread fragmented reading has become among Chinese university students but also show how its components are linked to students’ cognitive development. Specifically, the fragmentation of content, time, and attention in reading behavior has significant and positive effects on cognitive breadth. However, each of these components is somewhat negatively associated with cognitive depth.

Since the first e-book appeared in 1971, a succession of screen-based technologies has prompted people to adjust their approach to reading (Coiro, 2020). Fragmented reading results in a patchwork of information content with limited logical division or integration (He & Liu, 2018) which requires little time or attentiveness to absorb.

The descriptive statistics presented above demonstrated that many students read in this way. In particular, the mean values of each dimension indicate that students’ reading was notably fragmented across the temporal dimension. These results are consistent with Jiang (2017), who also reported that fragmented reading behavior was widespread, and highlighted its occurrence in fragments of time such as when waiting in line or before going to bed, etc (Jiang, 2017).

Despite recording the lowest means among the three dimensions of fragmented reading, fragmented attention was most strongly correlated with cognitive development in the present study. Information processing consumes attentional resources and students are less able to maintain their attention when exposed to continuous streams of fragmented information. Punctuated by countless pop-ups, the relentless flows of information that typify the new media age disrupt people’s thought processes and limit their attention spans (Xie, 2019). As a result, students in the new media age may be unable to allocate their attention to single tasks and instead engage in “mindless browsing” (Eagleton & Guinee, 2002). Habituated to this simplified mode of thinking and reading, students may oppose or fear traditional reading practices and even develop cognitive disorders (Guo, Kim & Rubin, 2014). The above findings corroborate previous conclusions that attentional fragmentation is a persistent and recurrent challenge to students in the new media age (Buchem & Hamelmann, 2010; Anobile, Stievano & Burr, 2013). Interestingly, research is yet to disclose any positive effects of attentional change on cognitive development.

The study results showed that fragmented reading was positively correlated with students’ cognitive breadth. Three factors may underpin this finding. First, easy access to information has become ubiquitous thanks to the Internet, which provides a vast range of reading resources, simplifying the process of obtaining interesting and regularly updated content. This link is confirmed by previous studies, which found that the opportunities for fragmented reading provided by the Internet motivate students to learn, increase their autonomy, improve the efficiency of their learning, and expand their horizons (Kua, Armitage & Branch, 2017). Second, fragmented reading permits students to access more information across a wider selection of texts. For example, when waiting for the bus or the airport, students can read a larger number of short articles or videos using their devices, ensuring that time is used efficiently and information can be obtained without difficulty (Li & Liu, 2019). In addition, compared with traditional reading methods, it is easy to locate diverse sources of information using Internet search engines. The fragmented approach does not require in-depth knowledge of a particular field but allows a broad perspective to be established on an area of knowledge. In other words, fragmented reading enables students to read relatively few words over a shorter period in order to “take a peek” at any professional or academic field. This form of reading thus weakens the boundaries between different domains of knowledge to a certain extent and expands the cognitive breadth of students, who can learn a little about a wider range of subjects.

However, the SEM results of the present study revealed that fragmented reading was negatively correlated with cognitive depth. One meta-analysis found that on-screen, fragmented reading may lead students to multitask while reading—to the detriment of their reading efficiency and comprehension of texts (Clinton-Lisell, 2021). Much earlier, Stoll commented that digital environments tend to encourage extensive, yet superficial, explorations of many topics (Stoll, 1995). However, reading is a multi-layered interactive process, requiring the reader to analyze text at different levels—including but not limited to superficial understanding (Brewer & Treyens, 1981). In addition, the reader should have preexisting knowledge of the subject to fully understand the text. However, convenient and simple, fragmentation encourages a somewhat mindless approach to texts, negatively impacting the development of students’ deep information-processing capacity and ability to sustain attention (Baron, 2015).

The contradictory findings of this study can be explained in various ways. Firstly, the availability of multiple forms of reading content on the Internet may restrict the developmental ability of college students to think independently, *i.e.,* encourage them to select information passively. It has been argued that the information processing ability of youths and young adults will adapt to the increasing volume of information they encounter (Shenk, 1999). However, when the amount of information exceeds a person’s processing capacity, their cognitive resources become overloaded, reducing their overall processing efficiency and slowing their development of cognitive depth (Shenk, 1999). According to this view, as students increasingly use mobile devices for these superficial interactions in the online world, they are likely to struggle with more challenging tasks (Duncan et al., 2016; Pfost, Dörfler & Artelt, 2013). This view is confirmed by the empirical conclusions of this study that the frequency of fragmented reading is negatively correlated with cognitive depth.

Moreover, students’ purposes for reading are often blurred by fragmentary reading. It is often the case that, attracted by some marginal content or hyperlinks on a web page, they will follow reading paths that deviate from the original reading track and forget the initial text. This is borne out by previous studies showing that the frequency of onscreen reading is associated with a decline in the quality of attention as technology becomes more integrated into people’s lives (Blumberg & Brooks, 2018), making deep immersion in a reading text increasingly challenging (Mangen & Kuiken, 2014). Moreover, advertisements on electronic devices are tailored to tempt and distract users, leading them to skip information rather than read it deeply and carefully (Baron, Calixte & Havewala, 2017), further exacerbating the trend toward attentional fragmentation and superficial learning. In the long term, attentional changes linked to fragmented reading increase the burden on students (Stern & Shalev, 2013) who browse rather than fully concentrate on what they are reading or thinking. Overall, fragmented reading harms the cognitive development of students, despite positive effects such as broadening their horizons.

The Organization for Economic Cooperation and Development (OECD)’s 2013 Skills Outlook pointed out that individuals’ cognitive level and information ability will have a profound impact on their economic and even social development (OECD, 2013). Fragmented reading helps people access large amounts of isolated and scattered information, improving their cognitive breadth, but because this information cannot be processed, coded, and organized promptly, it does not improve cognitive depth. This view is also held by Kravchenko (2021), who has claimed that large quantities of fragmented data and information cannot be actively processed and stored by students. As a result, fragmented reading cannot enact the crucial transition from cognitive breadth to cognitive depth.

Thus, the findings of this study contribute evidence to the debate on fragmented reading. Moreover, they provide empirical evidence for several hitherto arcane links between the constructs of fragmented reading and the cognitive development of students. Given these results and to optimize the development of students’ cognitive breadth and depth, the dual effects of fragmented reading need to be acknowledged and strategies to deal with these should be proposed.

## Conclusions

The present study has identified the three-dimensional structure of fragmentary reading which consists of fragmentation in the dimensions of content, time, and attention. It has rigorously investigated how these areas affected the cognitive development of Chinese university students.

The high mean values recorded in the survey corresponded to relatively high levels of fragmented reading, especially in its temporal form. These results indicate the importance of understanding the etiology and effects of engagement in fragmented reading among university students. The Pearson correlations showed that significant associations existed among all constructs, confirmed by the SEM results, which further indicated a strong positive association between fragmented reading and cognitive breadth, but also that this reading behavior was negatively correlated with cognitive depth.

The study results provide valuable insights for educators, mentors, and even students themselves. By collaborating to improve the accessibility and quality of fragmented information, governments, social organizations, and institutions of higher education can help students take their first steps towards understanding their subjects in depth. While fragmented reading provides a broad perspective on a domain of knowledge, the resulting information must be systematically classified and integrated with prior knowledge into mental maps that will guide a more profound understanding of the subject.

In the era of new media, where space, movement, and interactions are increasingly fluid, fragmented reading will become an unavoidable part of most students’ reading behavior. Switching from fragmented media to conventional book-reading requires time and experience: teachers must strive to develop the strengths of students and reduce their weaknesses to optimize the positive effects of fragmented reading on their cognitive development.

### Limitations and future studies

Although this quantitative study produced several noteworthy findings, it is crucial to acknowledge several limitations that future research may be able to address.

First and foremost, the results are of limited generalizability since they were derived from a sample of only 916 Chinese participants from six mainland universities. Future studies may extend and validate our findings on the effects of fragmented reading behaviors by recruiting a larger sample. Such studies might also investigate the nested relationships between individuals and groups who are particularly exposed to fragmented information. Moreover, the possible effects of individual and cultural differences on fragmentation and cognitive development can also be explored, since the current investigation was limited to a non-Western sample.

Secondarily, the current investigation only investigated five variables: the three constructs of fragmented reading and the two dimensions of cognitive development. Examining additional variables such as reading motivation and levels of reading engagement could yield interesting results, as could the inclusion of potential mediators such as personality traits and socioeconomic status. Previous research has already demonstrated that students with lower socioeconomic status are particularly prone to the negative effects of e-reading, whereas more privileged groups may be able to access advanced technologies to develop their cognitive levels (Zheng & Zheng, 2021), pointing to the possible presence of the Matthew effect on reading styles in the new media era: this remains an area for further empirical investigation.

##  Supplemental Information

10.7717/peerj.13861/supp-1 Supplemental Information 1 Raw Data

10.7717/peerj.13861/supp-2 Supplemental Information 2 Questionnaire (English and Chinese)

## Additional Information and Declarations

### Data Availability

The following information was supplied regarding data availability: Raw data is available in the Supplemental Files.
