# Supplementary material for: The effects of university students’ fragmented reading on cognitive development in the new media age: evidence from Chinese higher education
Source: PeerJ. 2022 Aug 23;10:e13861. doi: 10.7717/peerj.13861 (PMC9415515; doi:10.7717/peerj.13861)
Supplement: Supplemental Information 2 [file peerj-10-13861-s002.docx]

# 大学生新媒体阅读行为调查问卷

# Questionnaire on the new media reading behavior of university students

一、基本信息 Basic information

| **1.您的性别（ ）** A.男 B.女  **What's your gender?** **(…)** A. Male B. female |
| --- |
| **2.您所在的年级（ ）**A.大一 B.大二 C.大三 D.大四 E.研一 F.研二 G.研三  **What grade are you in? (…)**  I. **undergraduates**: A.1^st^ year; B.2^nd^ year; C.3^rd^ year D.4^th^ year  II. **postgraduates**: E.1^st^ year; F.2^nd^ year; G.3^rd^ year |
| **3.您的年龄：**_______  **Your age:**______ |
| **4.您当前所就读的大学属于（ ）**A.高职院校 B.私立高校 C.独立学院 D.省属普通本科院校 E.省属重点本科院校 F.部属重点院校  **The university you are currently attending is a (…)** A. Vocational college B. Private college/institute C. Autonomous college/institute D. Municipal university E. Provincial university F. National university |
| **5.您目前所读专业是_______________________（请填写专业名称的全称）**  **What is your current major________(please fill in the full name of the major)** |
| **6.过去一年，您的学习成绩在你们年级处于什么水平（ ）**A.前列 B.中上 C.中等 D.中下 E.后列  **In the past year, what is your academic performance level in your grade (…)**   1. Excellent B. Very good C. Good D. Average E. Below average |
| **7.您当前主要使用的手机是（ ）**A.安卓手机 B.苹果手机 C.其他  **What kind of mobile system are you using (…)** A. Android B. iPhone C. Other |

二、新媒体阅读中的碎片化行为 Fragmented behaviors in new media reading

**8. 根据自己的真实情况，请在下面相应方框内打✔：Please read the statements below and tick the appropriate box.**

| 碎片化行为题项  Items of fragmented reading behaviors | 非常不符合  Strongly  Disagree | 不太符合  Disagree | 不确定  Neutral | 比较符合  Agree | 非常符合  Strongly  Agree |
| --- | --- | --- | --- | --- | --- |
| A, **Content Fragmentation** |  |  |  |  |  |
| 8.1.每天我会频繁地打开新闻（微博、微信朋友圈等）读新信息  I frequently open news sites (Weibo, Wechat moments, etc.) to read new information every day. |  |  |  |  |  |
| 8.2.新闻（微博、微信等）推送什么我就读什么  I read whatever pops up in the news (Weibo, WeChat, etc.). |  |  |  |  |  |
| 8.3.我喜欢读文字比较少的内容（资讯），这样更省时省力  I like to read information with fewer words. It saves time and effort. |  |  |  |  |  |
| 8.4.当打开新闻（微博、微信朋友圈等）但没有新信息更新时，我会稍感失落  I feel a little frustrated when I open the news (Weibo, WeChat Moments, etc.) and no new information is updated. |  |  |  |  |  |
| 8.5.在跟别人聊天时，我好像什么都能懂一点，但大都懂的不深入  When chatting with others, I seem to understand everything a little, but most of content don't understand well |  |  |  |  |  |
| **8.6.当我看到感兴趣的某一信息时，我会去大量搜寻并阅读与之相关的信息  When I see a piece of information that interests me, I do a lot of searching and reading about it. |  |  |  |  |  |
| 8.7.我已经很久没有完整地读完一本书了  It's been a long time since I read a book in its entirety. |  |  |  |  |  |
| B, **Temporal Fragmentation** |  |  |  |  |  |
| 8.8.只要有时间，我就会刷手机信息（新闻、微博、微信朋友圈等）  As long as I have time, I check the information on my phone (News, Weibo, WeChat Moments, etc.). |  |  |  |  |  |
| 8.9.我觉得充分利用碎片化时间来刷手机信息帮我增长了不少见识  Making full use of brief interludes to check what pops up on my phone has helped me gain a lot of insight. |  |  |  |  |  |
| **8.10.当一篇新闻或文章较长而一次没有读完时，我会分几次，一定读完  When a piece of news or an article is too long to finish in one go, I carry on later and make sure I finish it. |  |  |  |  |  |
| 8.11.我很少有整块的时间（半天及以上）去查找、阅读某一方面的信息  I rarely have a long time (half a day or more) to find or read information on a particular subject. |  |  |  |  |  |
| 8.12.一会功夫不刷手机，我就感觉错过了好多重要信息  When I don't check my phone for a while, I feel like I'm missing a lot of important information. |  |  |  |  |  |
| C, **Attentional Fragmentation** |  |  |  |  |  |
| 8.13.我发现我现在很难长时间集中注意力去思考某问题  I find it hard to concentrate on something for a long time. |  |  |  |  |  |
| 8.14.我在自习（或听课）时，总是无意识地频繁查看手机  Without thinking, I frequently check my phone while studying (or in class). |  |  |  |  |  |
| 8.15.生活（或学习）上遇到什么难题，我第一反应是通过手机去查找解决办法  When I encounter troubles in life (or study), my first reaction is to find solutions on my mobile phone. |  |  |  |  |  |
| 8.16.出门忘带手机或手机（快）没电了，我会感到有些难捱，甚至有些惶恐  Leaving my phone behind or running out of battery makes me feel awkward and is even scary. |  |  |  |  |  |
| 8.17.我对网上出现的很多内容（如新的软件、网页等）都感兴趣，大都尝试一遍  I'm interested in a lot of things that come up on the Internet (new software, web pages, etc.). |  |  |  |  |  |
| 8.18.我的兴趣很多，但大都持续不了太久时间就变淡了  I have many interests, but most of them don't last very long and then fade away. |  |  |  |  |  |
| 8.19.很多次当我读某些信息过程中，会有种似曾相识的感觉，隐约记得以前读过  Many times when I read something, I get a sense of familiarity, a vague recollection of having read it before. |  |  |  |  |  |
| 8.20.对读过的很多内容，我有印象，但却难以逻辑清晰地梳理出来  I often have an impression of what I’ve read, but it can be hard to describe logically. |  |  |  |  |  |
| 8.21.我发现我的拖延症越来越严重了  I find my procrastination getting worse and worse. |  |  |  |  |  |
| 8.22.在决定做某事的过程中，我会经常陷入“两天打渔，三天晒网”的怪圈中  In the process of deciding to do something, I often fall into the trap of "two days fishing, three days drying nets." |  |  |  |  |  |

三、认知发展 Cognitive development

| 认知发展题项  Items of cognitive development | 非常不符合  Strongly  Disagree | 不太符合  Disagree | 不确定  Neutral | 比较符合  Agree | 非常符合  Strongly  Agree |
| --- | --- | --- | --- | --- | --- |
| D, **Cognitive Breadth** |  |  |  |  |  |
| 9.1新媒体（手机等）阅读让我的视野开阔了很多  New media (mobile phone, etc.) reading has broadened my horizons a lot. |  |  |  |  |  |
| 9.2我能够轻易地利用新媒体查询到专业相关的最新知识  I can easily use new media to find the latest knowledge related to my major. |  |  |  |  |  |
| 9.3我每天通过新媒体接触多种多样的信息，有一种紧跟潮流的满足感  Being exposed to so much information every day through new media gives me the satisfaction of being up to date. |  |  |  |  |  |
| 9.4我的阅读兴趣点（指阅读内容的主题）隔段时间就会发生变化  My reading interests (i.e., the reading content) change over time. |  |  |  |  |  |
| **9.5对与自己专业相关的知识，我兴趣浓厚  I am very interested in knowledge related to my major. |  |  |  |  |  |
| E, **Cognitive Depth** |  |  |  |  |  |
| *9.6虽然我通过新媒体了解了很多知识，但能够完全掌握的不多  Although I have learned a lot through new media, I couldn’t fully grasp it. |  |  |  |  |  |
| *9.7对于某一件事的看法，我很容易受网络（或他人）的影响  My opinion about a certain thing is easily influenced by the Internet (or other people). |  |  |  |  |  |
| *9.8对于每天阅读过的很多信息，我大都记忆不深刻  I don't remember much of what I read every day. |  |  |  |  |  |
| *9.9我很少主动就某些事件（或问题）的来龙去脉完全梳理清楚  I rarely take the initiative to fully clarify the context of an event (or problem). |  |  |  |  |  |
| **9.10我喜欢思考，对很多事有自己独到的见解  I like to think and have my own unique opinions on many things. |  |  |  |  |  |
| **9.11我喜欢坚持做某件事（或思考某个问题），跟自己较劲  I like to insist on doing or thinking about something, and compete with myself. |  |  |  |  |  |

*Reverse items

**Deleted items based on CFA
